# Poly (C)-Binding Protein 1 Regulates p63 Expression through mRNA Stability

**DOI:** 10.1371/journal.pone.0071724

**Published:** 2013-08-07

**Authors:** Seong-Jun Cho, Yong-Sam Jung, Xinbin Chen

**Affiliations:** Comparative Oncology Laboratory, School of Veterinary Medicine, University of California Davis, Davis, California, United States of America; University of Hawaii Cancer Center, United States of America

## Abstract

p63, a transcription factor and p53 family protein, plays a crucial role in tumor suppression and development of various epithelial tissues. While p63 expression is controlled mostly by post-translational modifications, recent studies indicate that transcriptional and posttranscriptional regulations are essential for proper p63 expression. Here, we investigated the regulation of p63 expression by poly (C)-binding protein 1 (PCBP1, also known as hnRNP-E1 and αCP1). We found that knockdown of PCBP1 decreases the level of p63 transcript and protein. We also found that PCBP1 regulates the stability of *p63* mRNA via binding to p63 3’UTR. Additionally, we found that a CU-rich element (CUE) in p63 3′UTR is bound by and responsive to PCBP1. Together, we conclude that PCBP1 regulates p63 expression via mRNA stability.

## Introduction

p63, a transcription factor and p53 family protein, is necessary for development of various epithelial tissues, stem cell maintenance, and tumor suppression [1]–[10]. The *p63* gene encodes two sets of isoforms: TAp63 and ΔNp63 [1]. TAp63 can transactivate a subset of p53 target genes such as *p21*, *Mdm2*, and *Bax*, and thus can regulate many cellular pathways as p53 [11]–[13]. ΔNp63 lacks the N-terminal activation domain conserved in p53, but carries an unique ΔN transactivation domain, which consists of the 14 unique residues plus the proline-rich domain in p63 [14]. Thus, ΔNp63 is capable of activating specific genes not induced by TAp63 isoforms [15], [16]. In addition, both *TAp63* and *ΔNp63* transcripts can be alternatively spliced at the 3′ end to generated at least 5 (α, β, γ, δ, and ε) isoforms [1].

p63 expression and activities are known to be controlled by post-translational modifications. For example, ubiquitin-proteasome-mediated degradation pathway regulated by E3 ligases, such as Pirh2 and Itch, controls p63 protein stability [17], [18]. p63 expression is also regulated by mRNA turnover or translation at post-transcriptional levels. The rate of mRNA turnover or translation is controlled by RNA-binding proteins, which bind to specific elements, such as AU-, CU-, or U-rich element, usually located in 5’ and/or 3’ UTRs of target mRNAs. Indeed, RNPC1 is found to regulate *p63* mRNA stability through directly binding to ARE and U-rich region in p63 3′UTR [19]. Moreover, HuR, an RNA-binding protein, is found to regulate ΔNp63 mRNA translation via binding to a U-rich element in p63 3′UTR [20].

In this study, we have investigated the regulation of *p63* mRNA stability by PCBP1, which is a member of the hnRNP K homologous domain protein family. As an RNA- or DNA-binding protein, PCBP1 regulates gene expression via a broad-spectrum of gene regulatory mechanisms, including transcription, mRNA splicing, mRNA stability, and translation [21]. Here, we found that knockdown of PCBP1 decreases the level of p63 transcript and protein. Moreover, we showed that knockdown of PCBP1 destabilizes *p63* mRNA via binding to its 3′UTR. Finally, we identified a CU-rich element (CUE) in p63 3′UTR, which is bound by and responsive to PCBP1. Together, we provided evidence that p63 expression is regulated by PCBP1 via mRNA stability.

## Materials and Methods

### Plasmids

pGEX vectors expressing GST-tagged PCBP1 were used for producing recombinant protein as previously described [22]. All lentivirus vectors (pLKO.1-puro) expressing shRNA of interest were purchased from Sigma. The targeting sequences are 5′-CGC TGA GTA CTT CGA AAT GTC-3′ for control luciferase shRNA, 5′- CCC ATG ATC CAA CTG TGT AAT -3′ for PCBP1 shRNA.

To generate the luciferase reporter under the control of *FOS* minimal promoter, a DNA fragment containing the *FOS* minimal promoter (−53 to +45 of the *FOS* promoter) was amplified using human genomic DNA with forward primer, FOS-F, and reverse primer, FOS-R. The PCR product was digested with *Kpn*I and *Hin*dIII and cloned into the pGL3-control vector (Promega), and the double-stranded oligonucleotide containing multi cloning site (*Xba*I; *Kpn*I; *Sac*I; *Mlu*I; *Nhe*I; *Xma*I; *Xho*I; *Bgl*II) was added into the 3′-end of the firefly luciferase gene coding region (CDS). The resulting vector named as pGL3-FSUT. To generate pGL3-FSUT/p63-WT, a DNA fragment containing the CUE (from 1903 to 2032 in p63-3′UTR) was amplified using human cDNA with forward primer, p63-1903-Xba-F, and reverse primer, p63-2032-Xho-R. The PCR product was digested with *Xba*I and *Xho*I and cloned into the pGL3-FSUT vector. To generate pGL3-FSUT/p63-CG, three-step PCR reactions were performed. The first-step was performed to separately amplify two cDNA Fragments. Fragment #1 was amplified with forward primer, p63-1903-Xba-F, and reverse primer, p63-2005-CG-R. Fragment #2 was amplified with forward primer, p63-2005-CG-F, and reverse primer, p63-2032-Xho-R. The second-step PCR reaction was performed to amplified fragment #3 using a mixture of fragment #1 and 2 as a template with primers, p63-1903-Xba-F and p63-2032-Xho-R. The third-step PCR reaction was performed using fragment #3 as a template with forward primer, p63-1903-CG-F, with reverse primer, p63-2032-Xho-R, and resulting fragment was cloned into pGL3-FSUT.

### Cell culture

Human pancreatic cancer cell line MIA PaCa2, human cervix cancer cell line ME180, and human breast cancer cell line MCF7 were obtained from the American Type Culture Collection (ATCC, Manassas, VA, USA). Human keratinocyte cell line HaCaT was obtained from CLS Cell line service (Eppelheim, Germany). Cell lines were cultured in DMEM (Invitrogen) supplemented with 10% fetal bovine serum (Hyclone) and maintained at 37°C in a humidified 5% CO_2_.

### RNA interference

For lentiviral shRNA transduction, a lentivirus vector (10 µg) expressing shRNA of interest, along with packaging plasmids, pRSV-REV (5 µg), pMDL g/p RRE (5 µg), and VSVG (5 µg), was cotransfected into 293T cells (8×10^6^) by using Expressfect transfection reagent (Denville Scientific) according to user’s manual. After 48 h, the supernatant containing shRNA-expressing lentivirus was harvested, filtered and concentrated by ultracentrifugation (25,000 rpm, 4°C, 2 h). The concentrated lentiviral particles were then used to transduce cells, followed by puromycin selection (1 µg/ml) for 3 days.

### Western blot analysis

Cells were cultured in various conditions and whole cell lysates were prepared by using 2X SDS sample buffer. Whole cell lysates were separated in 8∼12% SDS-PAGE, transferred to a nitrocellulose membrane, and incubated with primary and secondary antibodies, followed by enhanced chemiluminescent detection. The antibodies used in this study are anti-PCBP1 (E-2, Santa Cruz Biotech), anti-p63 (4A4, Santa Cruz Biotech), and anti-Actin (Sigma).

### RNA isolation, RT-PCR

Total RNA was isolated by using Trizol reagent (Invitrogen) according to user’s manual. cDNA was synthesized using MMLV reverse transcriptase (Promega) according to users’ manual. PCR was performed and separated by using 1.5∼2% agarose gel electrophoresis. The primer sequences for PCBP1, p63α, ΔNp63 and GAPDH, listed in Table 1.

**Table 1:** Primers for RT-PCR and cloning.

Name	Sequence
FOS-F	5′-GGGGGTACCCACTCATTCATAAAACGCTTG-3′
FOS-R	5′-GGGGAAGCTTGGCCGCCGGCTCAGTCTTGG-3′
p63-1903-Xba-F	5′-GGGGTCTAGAGCCTCACCATGTGAGCTCTT-3′
p63-2032-Xho-R	5′-GGGGCTCGAGTTCTCCTTCCCCTAAGAAATC-3′
p63-2005-CG-R	5′-CACCAACCCCACCACCTACCCACAACCCTTTGAAGATTAAGCAGG-3′
p63-2005-CG-F	5′-GGTTGTGGGTAGGTGGTGGGGTTGGTGTTGTCTGATTTCTTAGGGGAAG-3′
p63-1903-CG-F	5′-GGGGTCTAGAGCCTCACCATGTGAGCTCTTCCTATGGGTGTGGTAACTGCCAGCCCCCTAAAAG-3′
PCBP1-F	5′-GGCGGGTGTAAGATCAAAGA-3′
PCBP1-R	5′-GAGCGGAGAAATGGTGTGTT-3′
pre-p63-F	5′-ACGAAGATCCCCAGATGATG-3′
p63α-F	5′-GAGGTTGGGCTGTTCATCAT-3′
p63α-R	5′-GTGAATCGCACAGCATCAAT-3′
ΔNp63-F	5′-GGAAAACAATGCCCAGACTC-3′
ΔNp63-R	5′-TGGGGTCATCACCTTGATCT-3′
GAPDH-F	5′-AGCCTCAAGATCATCAGCAATG-3′
GAPDH-R	5′-ATGGACTGTGGTCATGAGTCCTT-3′

### RNA-immunoprecipitation (RNA-IP)

RNA-IP was carried out as previously described [23], [24]. Briefly, cells (8×10^6^) were lysed with 1 ml of lysis buffer (10 mM HEPES, pH 7.0, 100 mM KCl, 10 mM MgCl_2_, 0.5% NP-40, 1 mM DTT) supplemented with RiboLock Ribonuclease inhibitor (Fermentas) for 15 min on ice, and cell lysates were collected by centrifugation (13,000 rpm, 4°C, 10 min). The RNA-protein immunocomplexes were formed by incubating 0.4 ml of cell lysates with 2 µg of anti-PCBP1 or isotype control IgG at 4°C for 6 h and brought down by 20 µl of protein G bead (50% slurry). RT-PCR analysis was carried out to examine the RNA-protein interaction.

### Probe labeling and RNA Electrophoretic Mobility Shift Assay (REMSA)

All probes were labeled by *in vitro* transcription using a DNA fragment containing T7 promoter and various region of p63 3′UTR. Briefly, 500 ng of purified PCR product was incubated with 50 µCi of α-^32^P-UTP, 0.5 mM each of NTP (A, G, C), 20 unit of T7 RNA polymerase (Ambion) in 20 µl of reaction at 37°C for 1 h, followed by DNase I (1 unit) treatment for 15 min. The reaction mixture was purified by Sephadex G-50 column to remove unlabeled free nucleotide and the radioactivity of probes was measured by a scintillation counter. REMSA was carried out with a modified protocol as previously described [25]. Briefly, 250 nM of recombinant protein, 100 µg/ml of yeast tRNA, and 50,000 CPM ^32^P-labeled RNA probe were mixed in 20 µl of reaction buffer (10 mM Tris-Cl, pH 8.0, 25 mM KCl, 10 mM MgCl_2_, 1 mM DTT) at 25°C for 25 min. RNA/protein complexes were digested by adding 100 U RNase T1 at 37°C for 15 min and then separated in 7% of native PAGE gel. RNA-protein complexes were visualized by autoradiography.

### Luciferase assay

A dual-luciferase assay was performed as previously described [26].

### Statistical analysis

Statistical significance was determined by two-tailed Student’s *t*-test. *P*<0.05 was considered as significant.

## Results

### p63 expression is decreased by knockdown of PCBP1

**Figure 1:**
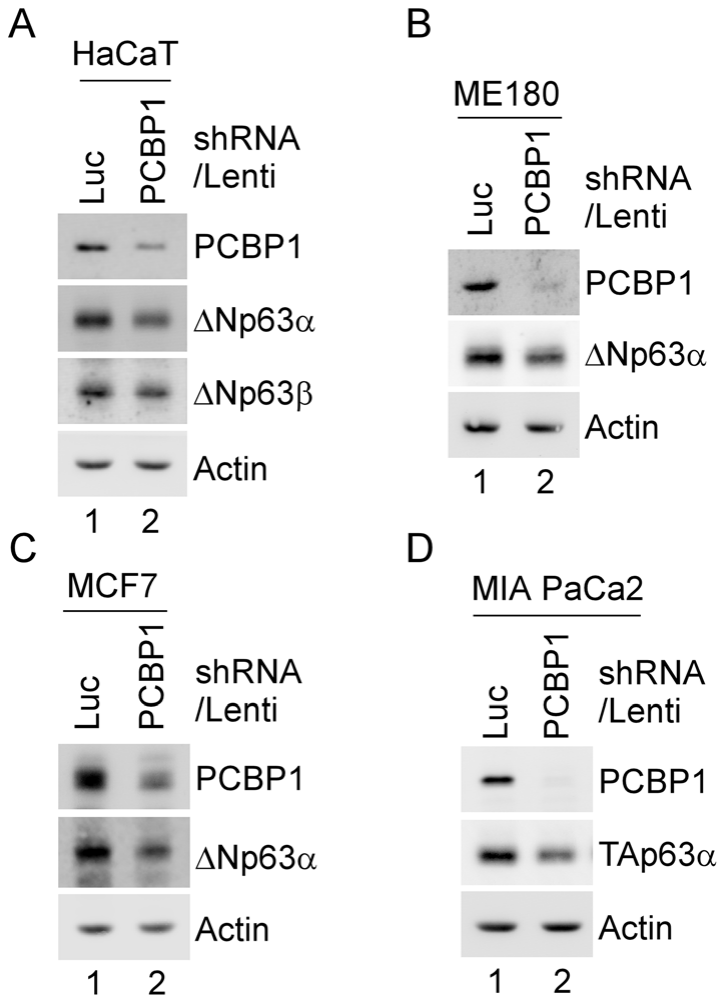
p63 expression is decreased by PCBP1-knockdown. HaCaT (A), ME180 (B), MCF7 (C), and MIA PaCa2 (D) cells were transduced with a lentivirus expressing a control luciferase (Luc) shRNA or PCBP1 shRNA, selected by puromycin for 3 d. Whole cell lysates were collected and the levels of PCBP1, Actin, ΔNp63α or ΔNp63β in HaCaT (A), ΔNp63α in ME180 and MCF7 (B–C), and TAp63α in MIA PaCa2 (D) cells were determined by Western blot analysis.

To determine whether PCBP1 regulates p63 expression, PCBP1 was knocked down in HaCaT, ME180, MCF7, and MIA PaCa2 cells transduced with a lentivirus expressing PCBP1 shRNA for 3 d. A lentivirus expressing luciferase shRNA was used as a control. We showed that the level of PCBP1 protein was decreased by PCBP1 shRNA, but not control shRNA (Fig. 1A–D, PCBP1 panel). Interestingly, we found that the levels of ΔNp63α and ΔNp63β proteins were markedly decreased upon knockdown of PCBP1 in HaCaT cells (Fig. 1A). Similarly, the level of ΔNp63α protein was decreased by knockdown of PCBP1 in ME180 and MCF7 cells (Fig. 1B–C). Moreover, we found that the level of TAp63α protein was decreased by knockdown of PCBP1 in MIA PaCa2 cells (Fig. 1D). Together, these data suggest that PCBP1 is necessary for proper expression of both TAp63 and ΔNp63.

### PCBP1 regulates p63 mRNA stability

**Figure 2:**
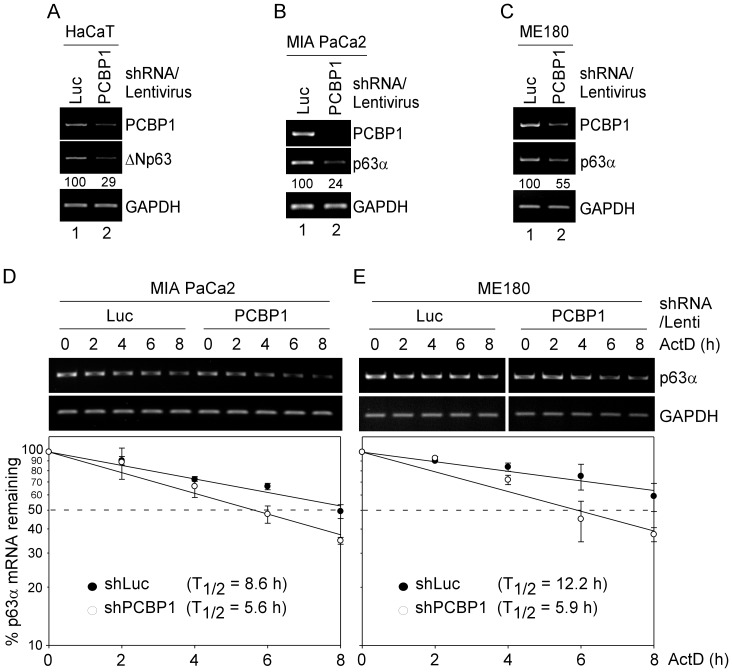
*p63* mRNA stability is regulated by PCBP1. (A–C) The level of *p63* transcript is decreased by PCBP1-knockdown. HaCaT (A), MIA PaCa2 (B), and ME180 (C) cells were transduced with a lentivirus expressing a control luciferase (Luc) shRNA or PCBP1 shRNA, selected by puromycin for 3 d. Total RNAs were isolated and RT-PCR was performed to measure the levels of *PCBP1*, *GAPDH*, *ΔNp63* (A), and *p63α* (B–C) transcript. (D–E) PCBP1-knockdown destabilizes *p63* transcript. MIA PaCa2 (D) and ME180 (E) cells were transduced with a lentivirus expressing a control luciferase (Luc) shRNA or PCBP1 shRNA, selected by puromycin for 3 d, followed by treatment with actinomycin D (5 µg/ml) for various times. The level of *p63α* and *GAPDH* transcript was measured by RT-PCR and quantified by densitometric analysis. The relative half-life of *p63α* transcript was calculated.

As an RNA-binding protein, PCBP1 is known to regulate gene expression via posttranscriptional mechanisms, including mRNA stability [21]. To explore how PCBP1 regulates p63 expression, RT-PCR was performed to measure the level of *p63* transcript in HaCaT, MIA PaCa2, and ME180 cells upon PCBP1-knockdown. We showed that the levels of *PCBP1* transcripts were decreased by PCBP1 shRNA, but not control shRNA in HaCaT, MIA PaCa2, and ME180 cells (Fig. 2A–C). Interestingly, PCBP1-knockdown decreased the level of *ΔNp63* transcript by 71% in HaCaT cells (Fig. 2A). Similarly, PCBP1-knockdown decreased the level of *p63α* transcript by 76% in MIA PaCa2 cells (Fig. 2B) and by 45% in ME180 cells (Fig. 2C). Since TAp63 and ΔNp63 are similarly regulated by PCBP1 (Fig 1), we focused on how PCBP1 regulates TAp63 and ΔNp63 expression via mRNA stability. To test this, the mRNA half-life of *p63α* transcripts was measured in MIA PaCa2 and ME180 cells transduced with a lentivirus expressing shRNA against luciferase or PCBP1 for 3 d, followed by treatment of actinomycin D to inhibit *de novo* RNA synthesis for various times. We found that the half-life of *p63α* mRNA was decreased by PCBP1-knockdown from ∼8.6 h to ∼5.6 h in MIA PaCa2 (Fig. 2D) and from ∼12.2 h to ∼5.9 h in ME180 cells (Fig. 2E), suggesting that PCBP1 is necessary for the maintenance of *p63α* mRNA stability. Indeed, a previous report showed that PCBP1 can function as a regulator of mRNA stability [21].

### PCBP1 directly binds to CUE in p63 3′UTR

**Figure 3:**
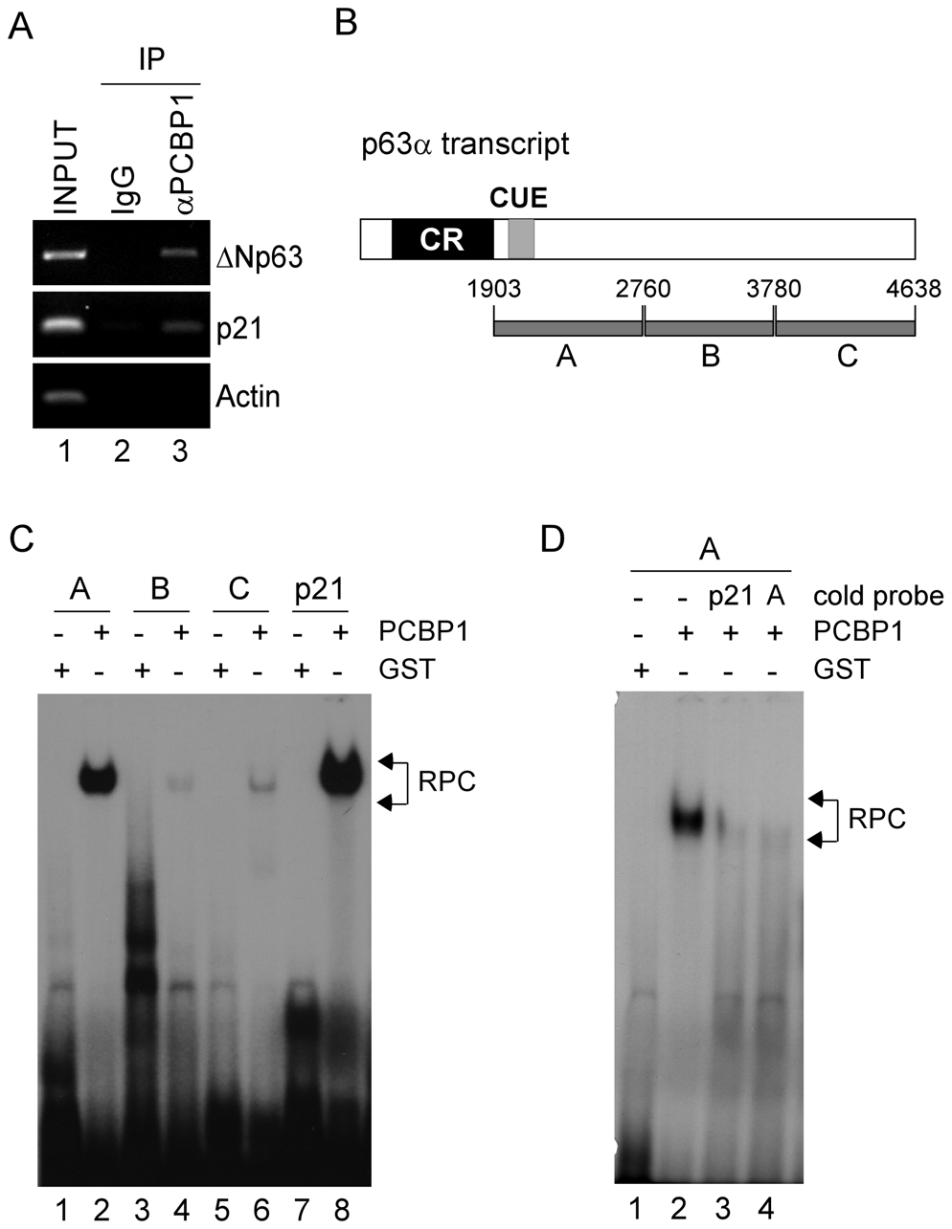
PCBP1 directly binds to p63 3′UTR . (A) PCBP1 interacts with *p63* transcripts *in vivo*. Cell lysates from HaCaT cells were collected and immunoprecipitated with PCBP1 antibody or control IgG, followed by RT-PCR analysis to determine the level of *ΔNp63* transcripts in control IgG and anti-PCBP1 immunocomplexes. The levels of *p21* and *Actin* transcripts were measured as positive and negative controls, respectively. (B) Schematic presentation of *p63α* transcript and the location of probes. CUE regions are shown in shaded box. (C) PCBP1 directly binds to p63 3′UTR. REMSA was performed by mixing a ^32^P-labeled RNA probes (A, B, C, or p21 probe) with recombinant GST or GST-fused PCBP1. The arrow indicates RNA-protein complexes. (D) REMSA assay was performed by mixing a ^32^P-labeled RNA probe A along with or without excess amount (50-fold) of unlabeled p21 or probe A.

To further explore how PCBP1 regulates *p63* mRNA stability, we determined whether PCBP1 is capable of binding to *p63* transcript. Thus, an RNA immunoprecipitation assay was performed using HaCaT cells. We found that *ΔNp63* mRNA was associated with anti-PCPB1, but not control IgG (Fig. 3A, ΔNp63 panel, compare lane 2 with 3). In addition, PCBP1 was found to associate with *p21* transcript (Fig. 3A, p21 panel), consistent with a previous report [27]. In contrast, no interaction was detected between *Actin* transcript and PCBP1 (Fig. 3A, Actin panel). Next, REMSA was performed to map the binding site of PCBP1 in *p63α* transcript with three p63α probes as indicated (Fig. 3B). p21 probe, which is derived from p21-3′UTR and known to contain a PCBP1-binding site [27], was used as a control. We showed that PCBP1 bound strongly to probe A or p21 probe, but weakly to probe B or C (Fig. 3C, compare lanes 1, 3, 5, and 7 with 2, 4, 6, and 8, respectively). The binding specificity of PCBP1 to probe A was examined by using an excess amount of cold p21 probe or probe A for a competition assay. We showed that the binding of PCBP1 to probe A was inhibited by cold probe A or p21 probe (Fig. 3D).

**Figure 4:**
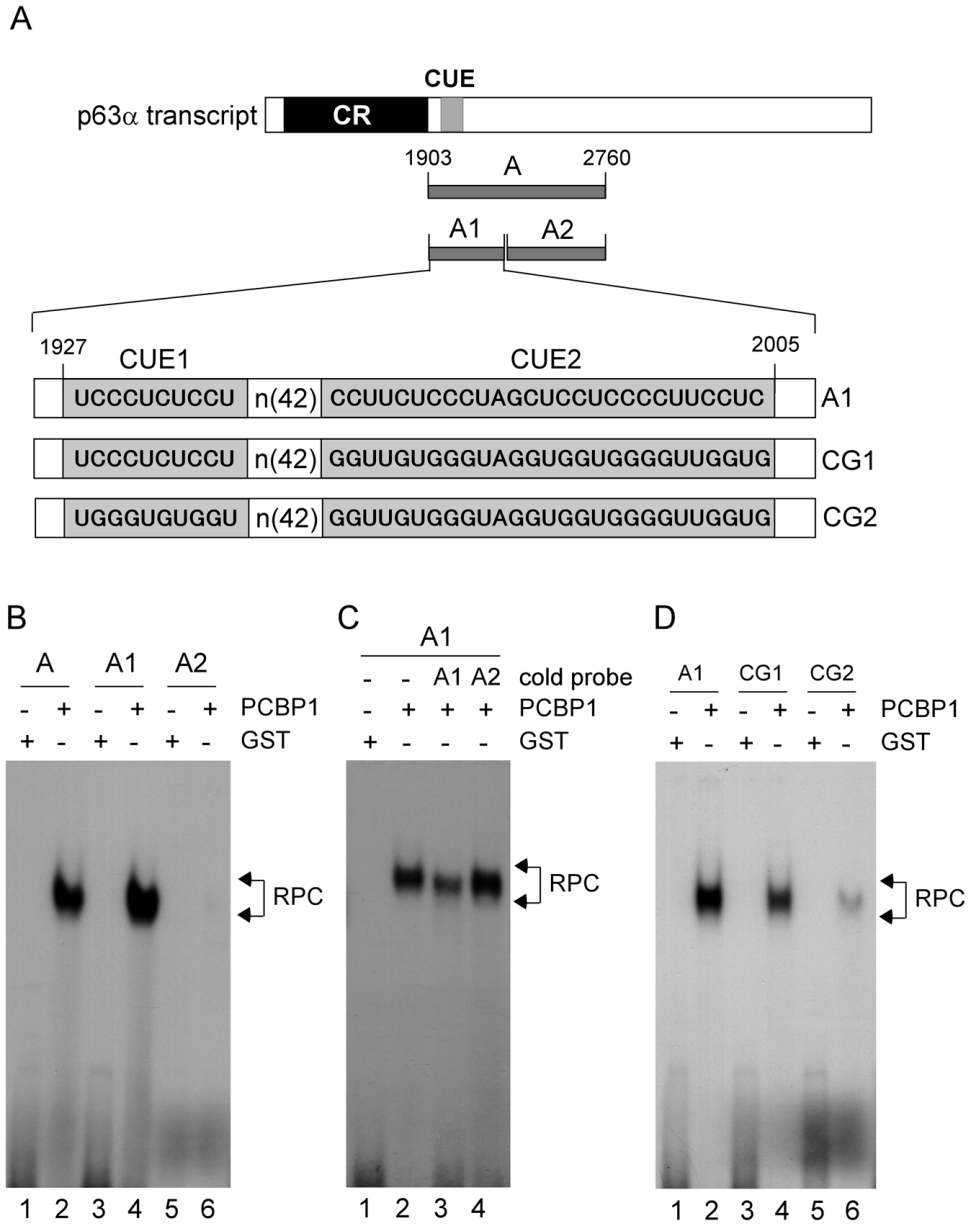
Identification of CUEs as a PCBP1-binding site in p63 3′UTR (A) Schematic presentation of *p63α* transcript and the location of probes. (B) REMSA was performed by mixing ^32^P-labeled RNA probes (A, A1, or A2) with recombinant GST and GST-PCBP1proteins, respectively. (C) REMSA assay was performed by mixing a ^32^P-labeled RNA probe A1 along with or without excess amount (50-fold) of unlabeled probe A1 or A2. (D) REMSA was performed by mixing a ^32^P-labeled RNA probe (A1, CG1, or CG2) with recombinant GST or GST-fused PCBP1.

To further map the PCBP1-binding region in probe A, two additional RNA probes were generated (Fig. 4A): probe A1, which is derived from nt 1903-2140 and contains two putative CUE and probe A2, which is derived from nt 2141-2760. We showed that PCBP1 bound to probe A1 as well as A, but not A2 (Fig. 4B, compare lanes 1, 3, and 5 with 2, 4, and 6, respectively). We also showed that the binding of PCBP1 to probe A1 was inhibited by cold probe A1, but not A2 (Fig. 4C, compare lane 2 with 3 and 4, respectively). To identify the PCBP1-binding element, we generated two mutant RNA probes (Fig. 4A): probe CG1, which carries mutations in CUE2; probe CG2, which carries mutation in both CUE1 and 2. We showed that the binding of PCBP1 to probe A1 was only slightly decreased by mutations in CUE2, but nearly abolished by mutations in both CUE1 and 2 (Fig. 4D, compare lanes 1, 3, and 5 with 2, 4, and 6, respectively). These data suggest that PCBP1 can bind to both CUE1 and 2, but CUE1 is sufficient for PCBP1 binding.

### The CUE in p63 3′UTR is responsive to PCBP1

**Figure 5:**
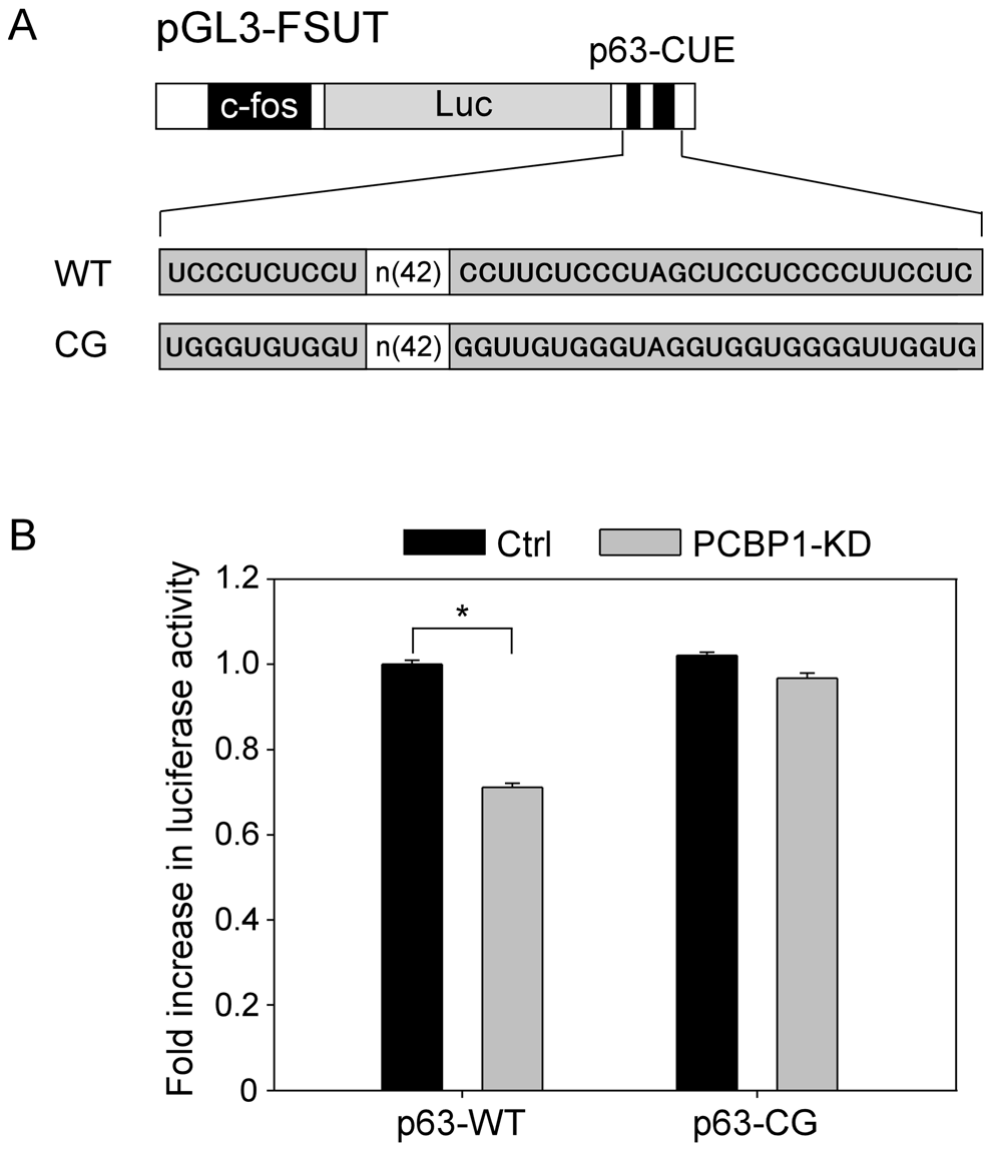
The CUE in p63 3′UTR is sufficient for PCBP1 to regulate p63 expression. (A) Schematic presentation of luciferase reporters that carry either wild-type p63-CUE or a mutated CUE (CG) derived from p63 3’UTR. (B) MCF7 cells were transduced with a lentivirus expressing control luciferase shRNA (Ctrl) or PCBP1 shRNA (PCBP1-KD) for 24 h, then transiently transfected with pGL3-FSUT/p63-WT or pGL3-FSUT/p63-CG for 48 h. Cell lysates were collected and the luciferase activity was measured. The experiment was performed in triplicate. Error bars indicate standard deviation. *P<0.05 by two-tailed *t*-test.

To determine whether the CUE is responsive to PCBP1, we generated two reporter vectors that carry luciferase coding region along with wild-type or a mutated CUE (CG) from p63 3′UTR (Fig. 5A). The reporters were expressed in MCF7 cells along with transduction with a lentivirus expressing a control or PCBP1 shRNA. We found that upon knockdown of PCBP1, the luciferase activity for the reporter containing wild-type p63-CUE was decreased to 71% of the control (Fig. 5B). However, knockdown of PCBP1 had no effect on luciferase activity for the reporter containing a mutated p63-CUE (Fig. 5B). The data suggest that PCBP1 binds to the CUE in p63 3′UTR and then regulates p63 expression via mRNA stability.

## Discussion

p63 shares many properties conserved in the p53 family [28]. p63 also has many distinct functions in tumor suppression, development and stem cell maintenance [29]. Thus, it is imperative to determine how p63 expression is differentially regulated by various pathways. Previously, we showed that RNPC1, a RNA binding protein and p53 family target, can modulate p63 expression via mRNA stability, but regulates p53 expression via mRNA translation [19], [30]. In addition, HuR, another RNA binding protein, positively regulates *p53* mRNA stability and translation, but is only found to regulates *p63* mRNA translation [20], [31], [32]. In this study, we showed that PCBP1 can directly bind to *p63* mRNA via CU-rich element in 3′UTR and the CUE is responsive to PCBP1. In addition, we showed that PCBP1 regulates p63 expression via mRNA stability. However, it remains unclear whether and how PCBP1 regulates p53 expression.

In this study, we identified a well-conserved CUE at a defined region in p63 3’UTR. Moreover, the CUE is sufficient for PCBP1-binding and for regulating *p63* mRNA stability. However, the mechanism by which PCBP1 regulates *p63* mRNA stability remains unclear. One possibility is the recruitment of α-complex for regulating *p63* mRNA stability. α-complex is comprised of PCBP1, PCBP2, AUF1, and poly(A)-binding protein (so called as PABP-C) and known to bind CUE in α-globin 3′UTR [21]. In addition, α-complex regulates *α-globin* mRNA stability through PABP-mediated inhibition of endoribonuclease [33], [34]. The other possibility is inhibition of negative regulators for mRNA stability, such as miRNA-203 [35]. If PCBP1 and its complex, such as α-complex, occupy a region of p63 3′UTR, the binding of a negative regulator for mRNA stability might be inhibited. Thus, future studies are warranted to explore how PCBP1 regulates *p63* mRNA stability by cooperating or competing with other RNA-binding proteins and miRNAs.

PCBP1 regulates gene expression as transcriptional and post-transcriptional regulators [21]. Thus, how PCBP1 is functionally correlated with p63? Recently, p63 is known to modulate tubular formation via epithelial-to-mesenchymal transition (EMT) through growth suppressing genes, including *p21*, *PUMA*, and *MIC-1* [36]. In addition, PCBP1 modulates transforming growth factor-β-induced EMT through translational regulation of disabled-2 (Dab2) and interleukin-like MET inducer (ILEI) [37]. These studies indicate that both PCBP1 and p63 play a role in a signaling axis for EMT. Considering that uncontrolled regulation of EMT causes a variety of diseases, including fibrosis and metastatic progression of tumors, further studies to address the linkage between PCBP1 and p63 would provide an avenue for anticancer therapeutic strategies.
